# Peripartum Cardiomyopathy-Induced Cardiogenic Shock Causing Hypoxic-Ischemic Encephalopathy in a COVID-19 Patient

**DOI:** 10.1155/2022/7114732

**Published:** 2022-11-24

**Authors:** Minh Khoi Le, Thanh Hieu Nguyen

**Affiliations:** ^1^Cardiovascular Center, University Medical Center, Ho Chi Minh City, Vietnam; ^2^Department of Critical Care Medicine, University of Medicine and Pharmacy at Ho Chi Minh City, Ho Chi Minh City, Vietnam; ^3^Department of Anesthesia, University Medical Center, Ho Chi Minh City, Vietnam

## Abstract

**Background:**

Cardiogenic shock complicating peripartum cardiomyopathy (PPCM) is a rare but lethal syndrome. The etiology of PPCM is not fully elucidated and is probably multifactorial, and viral infection might play some role. It has been documented that the severe acute respiratory syndrome coronavirus 2 (SARS-CoV-2) directly invades the cardiomyocytes and most commonly damages this vital organ via complex systemic devastating mechanisms. *Case presentation*. A 28-year-old pregnant female was admitted to a COVID-19 field hospital due to a SARS-CoV-2 infection. She gave birth by spontaneous vaginal delivery at 34 gestational weeks. Six hours after the delivery, she presented signs of hemodynamic collapse and became comatosed, requiring a transfer to the COVID-19 intensive care center. The brain magnetic resonance imaging excluded thromboembolism, intracerebral hemorrhage, and central nervous system infection and revealed a hypoxic-ischemic encephalopathy. Bedside echocardiography documented a dilated left ventricle and severely reduced left ventricular systolic function with an ejection fraction of 24%. The management was aimed at a cardiogenic shock secondary to peripartum cardiomyopathy. The clinical course was favorable: the hemodynamics stabilized, the cognitive function fully recovered, and the patient was extubated on the second day of admission to the intensive care unit. The patient was discharged from the hospital ten days after admission. Neurological and cardiovascular checkups six months after discharge showed full recovery.

**Conclusion:**

Peripartum cardiomyopathy-induced cardiogenic shock with severe neurological consequences in COVID-19 patients was rare but did exist. A systemic approach and vigorous efforts to pinpoint the accurate diagnosis played important roles in the prompt and appropriate management.

## 1. Background

The outbreak of the coronavirus disease 2019 (COVID-19) pandemic has become one of the most serious health crises in human history [1]. In addition to chronic comorbidities such as diabetes, cardiovascular diseases, chronic kidney disease, and obesity, pregnancy has been increasingly recognized as a risk factor of severity in patients contracting SARS-CoV-2 [2]. The physiological changes during pregnancy, such as altered immune system and elevation of the diaphragm leading to reduced functional residual capacity, make pregnant women more vulnerable to COVID-19. COVID-19-related cardiomyopathy has been well reported early at the beginning of the pandemic. In a report of general COVID-19 patients in Washington State, 33% of those who required admission to the intensive care unit developed cardiomyopathy [3]. Cardiac damage may be caused by direct viral invasion or secondary to multiple pathological events in severe SARS-CoV-2 infection [4–6]. Although there have been sporadic reports on COVID-19-related cardiomyopathy in pregnant women, our knowledge in this area is still limited.

Cardiogenic shock complicating peripartum cardiomyopathy is a rare but lethal syndrome [7]. In this paper, we report the successful management of cardiogenic shock secondary to a COVID-19-related cardiomyopathy in a postpartum patient.

## 2. Case Presentation

A 28-year-old female gave birth by spontaneous vaginal delivery at 34 gestational weeks to her second child in a COVID-19 field hospital, where she had been admitted for her SARS-CoV-2 infection. Six hours after the delivery, she presented the first signs of hemodynamic instability and reduced consciousness. Her arterial blood pressure steadily deteriorated, requiring the intravenous norepinephrine infusion. After a quick online consultation, she was transferred to our tertiary COVID-19 intensive care center. Upon arrival at our center, her Glasgow coma scale score was 8, dictating immediate intubation. She was hemodynamically supported with intravenous norepinephrine infusion at 0.2 mcg/kg/minute. Her arterial blood pressure was 115/65 mmHg with sinus tachycardia at 105 beats per minute (bpm) and SpO_2_ 95% on FiO_2_ 0.6. Her ECG showed no apparent abnormality except sinus tachycardia. Bedside echocardiography demonstrated global cardiac hypokinesia, a dilated left ventricle with the left ventricular diastolic diameter (LVIDd) of 60 mm, and a severely impaired left ventricular systolic function with a left ventricular ejection fraction (LVEF) of 24%. The reverse transcription-polymerase chain reaction for SARS-CoV-2 was positive, confirming the diagnosis of COVID-19. The emergency head computed tomography scan was normal without a sign of ischemia or intracerebral hemorrhage.

**Figure 1:**
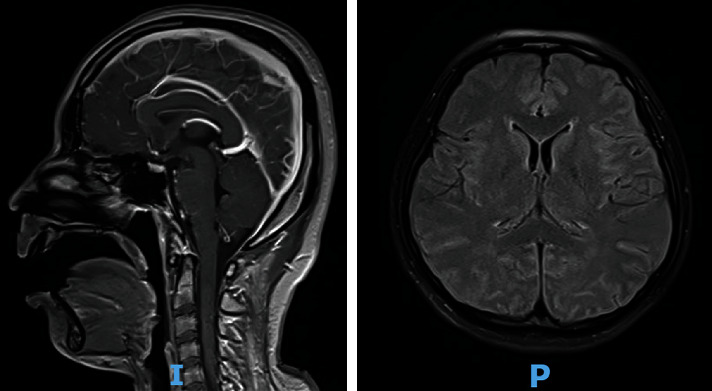
Brain magnetic resonance imaging on the same day of admission to the intensive care unit showed diffuse brain edema and hyperintensity of the bilateral sulcus, gyrus and basal ganglia on fluid-attenuated inversion recovery (FLAIR), and mildly contrast enhancement of the meninge, compatible with hypoxic-ischemic encephalopathy.

**Figure 2:**
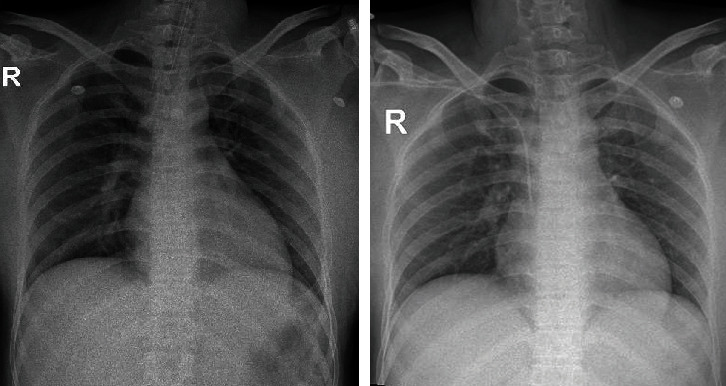
Chest X-ray images on admission (left) and postextubation (right) showed bilateral ground glass opacities consistent with mild COVID-19.

The subsequent magnetic resonance imaging (MRI) documented evidence of a hypoxic brain injury and excluded microthrombus, vasculitis, and central nervous system infection (Figure 1). Her chest X-ray (Figure 2) and arterial blood gas showed no criteria of acute respiratory distress syndrome (pH 7.46, pCO2 34 mmHg, pO_2_ 221 mmHg, HCO_3_ 24 mmol/L, and a P/F ratio 548). Her detailed laboratory results on admission are presented in Table 1.

She was diagnosed with hypoxic-ischemic encephalopathy secondary to cardiogenic shock caused by COVID-19-related peripartum cardiomyopathy. The intravenous dobutamine was added as an inotropic agent while norepinephrine was gradually reduced, and intravenous furosemide was started in addition to institutional standard management of COVID-19 patients.

The day after admission, the patient regained her full consciousness and showed no residual neurological deficit. Bedside echocardiography showed significant improvement in contraction with an LVEF of 45%. Troponin values remained stable, ranging from 224 to 262 ng/L. Serum NT-proBNP decreased from 689 to 390 pg/mL. She was extubated on the second day. The post-extubation chest X-ray was reassured, and dobutamine was gradually tapered off on the third day. The patient was discharged from the hospital ten days after admission. Her baby was discharged from the neonatal unit two days later in good health condition.

**Figure 3:**
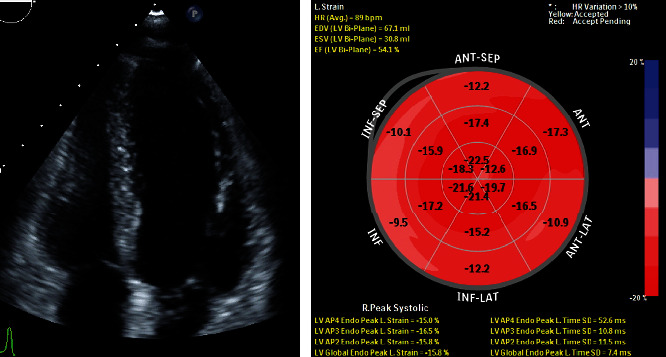
Echocardiographic examination at 6 months after patient's discharge from hospital.

The follow-up examination six months after her discharge from the hospital detected no neurologic deficits nor signs of heart failure. The echocardiogram noted normal cardiac morphology, no cardiac chamber dilatation with LVIDd of 49 mm, good diastolic and systolic functions with an LVEF of 55%, and a mild decrease in LV global longitudinal strain (-15.8%) (Figure 3). Serum troponin T was 11.9 ng/L and troponin I was 2.3 ng/L.

## 3. Discussion

Although respiratory manifestations are the hallmark of COVID-19, the disease is not confined to the lungs. SARS-CoV-2 can be detected in multiple organs, including the lungs, pharynx, heart, liver, brain, and kidneys [8, 9]. An early study reported a few interstitial mononuclear inflammatory infiltrates causing no significant damage to the heart tissue [10]. Pregnant females contracting SARS-CoV-2 have poorer outcomes [2, 5]. Two cases of COVID-19-related cardiomyopathy in pregnancy have been reported, but the cardiac function was moderately reduced with an LVEF of 40%-45% [6]. Herein, we reported a case of severe cardiac dysfunction leading to cardiogenic shock and hypoxic-ischemic encephalopathy in a woman infected with SARS-CoV-2.

In this patient, we could not exclude with certainty the possibility of septic shock in the context of increased WBC and serum procalcitonin. However, the rapid resolution of shock and complete neurological recovery made the diagnosis less probable. The most likely diagnosis is peripartum cardiomyopathy (PPCM), a rare form of pregnancy-associated cardiac dysfunction. The European Society of Cardiology defines PPCM as heart failure secondary to LV systolic dysfunction sometime before and several months after delivery. Echocardiography is the imaging modality of choice [11]. We reported previously that the combination of echocardiography and lung ultrasound could help decipher the mechanism of cardiogenic shock [12]. The etiology of PPCM is not fully elucidated and is probably multifactorial [13]. Presumable mechanisms for the development of PPCM might include nutritional deficiencies, autoimmune processes, and viral myocarditis [14]. In this case, it could hypothesize that the SARS-CoV-2-induced myocarditis may facilitate or even trigger the development of PPCM.

The recommended management of PPCM with severe acute heart failure or cardiogenic shock includes optimizing preload, respiratory support to optimize oxygenation, using inotropes and/or vasopressors, urgent delivery, and considering mechanical circulatory support if necessary [11]. We treated this patient as PPCM with intravenous dobutamine, diuretics, respiratory support, and tapered norepinephrine, the main vasopressor used in septic shock. The favorable response to the treatment supported the diagnosis of PPCM.

In general, recovery of cardiac function from PPCM is greater than that in men and nonperipartum cardiomyopathy, and recovery frequently occurs within 3 to 6 months [15, 16]. Our patient showed normal physical activity and cognitive function. Cardiac checkups, especially cardiac enzyme, heart chamber size, and systolic and diastolic function, as well as LV global longitudinal strain, were all in the normal range indicating a complete cardiac recovery.

## 4. Conclusion

We reported a case of peripartum cardiomyopathy in a COVID-19 patient with cardiogenic shock and hypoxic-ischemic encephalopathy. With intense effort to reach the most probable diagnosis, we could rapidly pinpoint the severe cardiac dysfunction as the cause of hemodynamic instability in otherwise not critical lung damage. The management was adjusted appropriately, and the patient quickly and completely recovered, although the initial presentations suggested an imminent peril. The peripartum cardiomyopathy in a COVID-19 patient is not common, and the peripartum cardiomyopathy-induced cardiogenic shock with hypoxic-ischemic encephalopathy is even rarer, but it does exist. Bedside echocardiography proves a useful imaging modality in screening, diagnosis, and monitoring the response to management and postdischarge follow-up.

## Figures and Tables

**Table 1:** Patient's laboratory results upon admission to the COVID-19 intensive care unit.

Parameters	Values	Parameters	Values
WBC (G/L)	16,96	BNP (pg/mL)	689
Hb (g/L)	91.5	Troponin T (ng/L)	224
Potassium (mmol/L)	4.0	Serum NH3 (g/dL)	77
CRP (mg/L)	43.9	PCT (ng/mL)	9.2
Fibrinogen (g/L)	3.80	D-dimer (mg/mL)	1269
Ferritine (ng/mL)	182.68	Interleukin-6 (pg/mL)	102.42

## Data Availability

The essential data are provided in the manuscript, and there is no additional data or material.

## Abbreviations

COVID-19: Coronavirus disease 2019
LVEF: Left ventricular ejection fraction
LVIDd: Internal diameter in diastole
MRI: Magnetic resonance imaging
NT-proBNP: N-terminal proB-type natriuretic peptide
PPCM: Peripartum cardiomyopathy
SARS-CoV-2: Severe acute respiratory syndrome coronavirus 2.
